# Phosphate control in reducing FGF23 levels in hemodialysis patients

**DOI:** 10.1371/journal.pone.0201537

**Published:** 2018-08-07

**Authors:** Cristian Rodelo-Haad, Maria E. Rodríguez-Ortiz, Alejandro Martin-Malo, M. Victoria Pendon-Ruiz de Mier, M. Luisa Agüera, Juan R. Muñoz-Castañeda, Sagrario Soriano, Francisco Caravaca, M. Antonia Alvarez-Lara, Arnold Felsenfeld, Pedro Aljama, Mariano Rodriguez

**Affiliations:** 1 Instituto Maimónides de Investigación Biomédica de Córdoba (IMIBIC), Reina Sofia University Hospital, University of Cordoba, Cordoba, Spain; 2 Nephrology Service, Reina Sofia University Hospital, Cordoba, Spain; 3 RETICs-REDinREN (National Institute of Health Carlos III), Madrid, Spain; 4 Nephrology Service, Infanta Cristina Hospital, Badajoz, Spain; 5 Wadsworth VA, UCLA, Department of Medicine, Veterans Affairs Greater Los Angeles Healthcare System and the David Geffen School of Medicine, University of California Los Angeles, Los Angeles, California; University of Milan, ITALY

## Abstract

**Background:**

In hemodialysis patients, high levels of Fibroblast Growth Factor 23 (FGF23) predict mortality. Our study was designed to test whether the control of serum phosphate is associated with a reduction in serum FGF23 levels. Additionally other variables with a potential effect on FGF23 levels were evaluated.

**Material and methods:**

The effect of sustained (40-weeks) control of serum phosphate on FGF23 levels (intact and c-terminal) was evaluated in 21 stable hemodialysis patients that were not receiving calcimimetics or active vitamin D. Patients received non-calcium phosphate binders to maintain serum phosphate below 4.5 mg/dl. In an additional analysis, values of intact-FGF23 (iFGF23) and c-terminal FGF23 (cFGF23) from 150 hemodialysis patients were correlated with parameters of mineral metabolism and inflammation. Linear mixed models and linear regression were performed to evaluate longitudinal trajectories of variables and the association between FGF23 and the other variables examined.

**Results:**

During the 40-week treatment, 12 of 21 patients achieved the target of serum phosphate <4.5 mg/dl. In these 12 patients, iFGF23 decreased to less than half whereas cFGF23 did not reduce significantly. In patients with serum phosphate >4.5 mg, iFGF23 and cFGF23 increased two and four-fold respectively as compared with baseline. Furthermore, changes in serum phosphate correlated with changes in C-reactive protein (*hs*-CRP). In our 150 hemodialysis patients, those in the higher tertile of serum phosphate also showed increased *hs*-CRP, iPTH, iFGF23 and cFGF23. Multiple regression analysis revealed that iFGF23 levels directly correlated with both serum phosphate and calcium, whereas cFGF23 correlated with serum phosphate and *hs*-CRP but not with calcium.

**Conclusions:**

The control of serum phosphate reduced iFGF23. This reduction was also associated with a decreased in inflammatory parameters. Considering the entire cohort of hemodialysis patients, iFGF23 levels correlated directly with serum phosphate levels and also correlated inversely with serum calcium concentration. The levels of cFGF23 were closely related to serum phosphate and parameters of inflammation.

## Introduction

Fibroblast Growth Factor 23 (FGF23) is a hormone secreted by osteocytes and mature osteoblasts. FGF23 enhances urinary phosphate excretion and reduces renal production of 1,25 dihydroxy-vitamin D (1,25 (OH)_2_ D) [1–3]. In chronic kidney disease (CKD), FGF23 is increased in early stages of renal disease even before phosphate retention occurs [4–7]. Furthermore, FGF23 is known to inhibit parathyroid hormone (PTH) secretion, but in CKD FGF23 fails to inhibit PTH secretion due to reduced expression of FGFR1 and Klotho in uremic hyperplastic parathyroid glands [8]. Conversely, PTH stimulates FGF23 production [9]. Recent studies have shown that low serum calcium (Ca) may be associated with low FGF23 production [10–12]. However, cell-sensing mechanisms triggering FGF23 production and secretion have not been fully characterized. Some studies have failed to demonstrate significant changes in serum FGF23 levels following short-term control of serum phosphate, calcium or PTH [13,14]. Furthermore, the level of complexity of FGF23 regulation has further increased because of recent evidence that both inflammation and iron status also influence the production of FGF23 [15–18]. Moreover, there are circulating fragments of c-terminal and intact FGF23, both of which are elevated in hemodialysis patients. However, it is not clear whether both molecules have the same clinical significance.

A matter of concern for clinicians is that high levels of FGF23 are strongly associated with cardiovascular disease (CVD), and an independent predictor of mortality [19–22]. Thus, control of FGF23 is likely a major issue in clinical practice.

In patients with CKD, studies have shown that the control of serum phosphate using oral phosphate binders can reduced serum FGF23 levels [23–25]. The effect of the control of serum phosphate on FGF23 in dialysis patients has not been sufficiently evaluated. Some studies have reported a reduction of FGF23 after control of serum phosphate [26–30], however, other studies have not reported the same effect [31]. Moreover, it is unknown to what extent a control of phosphate below the upper limit of normal may affect the levels of intact and/or c-terminal FGF23 in hemodialysis patients. Finally, it is also unknown whether inflammation mitigates the potential reduction in FGF23 obtained by hyperphosphatemia control.

The goal of the present study was to evaluate in stable hemodialysis patients, the effect of long-term control of serum phosphate concentration on FGF23 levels (both c-terminal and intact molecule). Our study also aimed to evaluate whether other factors besides phosphate, such as inflammation, calcium and PTH are associated with high serum concentrations of the two FGF23 molecules so they could be used as potential targets to reduce the levels of FGF23

## Materials and methods

### The effect of long-term control of serum phosphate concentration on FGF23 levels

#### Patients

The effect of long-term (40 weeks) control of serum phosphate on FGF23 levels was tested in a group of 21 hemodialysis patients (S1 Dataset). Patients were selected from a total of 150 clinically stable HD patients (S2 Dataset). A detailed description of the demographic, biochemical data and treatment characteristics of the 21 selected as well as the 150 patients is shown in Table 1.

**Table 1:** Demographic, clinical and biochemical characteristics of the 21 patients included in the longitudinal analysis and the cross-sectional study (*n* = 150).

Variable	*n* = 21	*n* = 150
**Age (years)** ^**§**^	70.0 (62.0–76.5)	71.0 (58.7–81.0)
**BMI** ^**a**^^,^ ^**¶**^	26.3 (23.1–30.2)	26.1 (22.1–30.2)
**Gender (n, %)** *Male* *Female*	12 (57.1) 9 (42.9)	85 (56.7) 65 (43.3)
**Renal disease**		
*Unknown (%)*	38.1	36.0
*Diabetes (%)*	14.3	18.0
*Hypertension (%)*	4.8	5.3
*Glomerulonephritis (%)*	14.3	10.7
*Polycystic (%)*	9.5	14.0
*Others (%)*	19.0	16.0
**Comorbidities**		
*Hypertension (%)*	71.4	83.3
*Diabetes (%)*	23.8	29.3
*Coronary artery disease (%)*	23.8	21.3
*Cerebrovascular disease (%)*	14.3	12.7
***Charlson Comorbidity index*** ^**§**^	4.0 (3.0–5.0)	4.0 (2.0–5.0)
**Vascular Access**		
*AV Fistula*, *n (%)*	11 (52.4)	102 (68.0)
*Catheters*, *n (%)*	6 (28.6)	41 (27.3)
*Grafts*, *n (%)*	4 (19)	7 (4.7)
**Dialysate Calcium 3 mEq/L (n, %)**	21 (100)	131 (87.3)
**Dialysis technique** *HF-HD (n*, *%)* *OL-HDF (n*, *%)*	21 (100) 0	69 (46) 81 (54)
**Dialysis vintage (months)** ^**b**^^,^ ^**§**^	53.7 (33.7–83.8)	50.1 (17.5–82.1)
**Dialysis Duration (min)** ^**§**^	245.0 (243.0–250.0)	250.0 (240.0–250.0)
**KtV** ^**§**^	2.0 (1.8–2.4)	1.9 (1.7–2.3)
**Kt (L/session)** ^**§**^	60.1 (48.8–66.65)	59.8 (52.0–64.0)
**Albumin (g/L)**	3.6 (3.3–3.8)	3.7 (3.4–3.9)
**Hb (g/L)** ^**c**^^,^ ^**§**^	11.2 (10.4–11.9)	11.2 (10.4–12.0)
**TSAT (%)**^**d**^^,^ ^**§**^	29.0 (21.5–37.5)	26.0 (21.0–34.0)
**Ferritin (ng/dl)** ^**§**^	529.0 (351.5–757.0)	468.5 (327.0–742.2)
***hs-*CRP (mg/L)** ^**e**^^,^ ^**§**^	8.0 (4.1–11.4)	7.2 (3.5–10.8)
**Ca (mg/dL)** ^**e**^^,^ ^**§**^	8.9 (8.5–9.3)	8.8 (8.4–9.2)
**iCa (mEq/L)** ^**f**^^,^ ^**§**^	2.2 (2.1–2.2)	2.2 (2.1–2.3)
**P (mg/dl)** ^**g**^^,^ ^**§**^	4.3 (3.6–5.5)	4.3 (3.7–5.3)
**Alkaline phosphatase (U/L)** ^**§**^	85.5 (74.0–108.7)	90.5 (71.0–121.0)
**iPTH (pg/ml)** ^**h**^^,^ ^**§**^	284.0 (199.5–445.0)	263.0 (151.7–434.5)
**25(OH)D (ng/ml)** ^**i**^^,^ ^**§**^^, *^	9.2 (8.5–18.)	8.1 (6.9–10.5)
**1,25 (OH)**_**2**_ **D (pg/ml)** ^**j**^^,^ ^**§**^^, *^	9.2 (7.7–10.3)	11.0 (5.0–14.8)
**iFGF23 (pg/ml)** ^**k**^^,^ ^**§**^	614.0 (346.0–958.5)	502.5 (167.0–1224.5)
**cFGF23 (RU/ml)** ^**l**^^,^ ^**§**^	880.0 (547.5–1443.5)	900.5 (400.2–1819.7)
**Phosphate Binders (n, %)**	21 (100)	67 (44.7)
**Paricalcitol (n, %)**	——	41 (27.3)
**Cinacalcet (n, %)**	——	20 (13.3)

^¶^ Mean ± Standard deviation (SD)

^§^ Median and Interquartile Range (IQR)

^a^ BMI, Body Mass Index

^b^ Dialysis Vintage, Time since the initiation of dialysis

^c^ Hb, Hemoglobin

^d^ TSAT, Transferrin Saturation

^e^ *hs*-CRP, C Reactive Protein

^f^ iCa, Ionized Serum Calcium

^g^ P, Serum Phosphate

^h^ PTH, Intact Parathyroid Hormone

^i^ 25 (OH)D, 25 hydroxy vitamin D (calcidiol)

^j^ 1,25 (OH)_2_ D, 1,25 dihydroxy vitamin D (calcitriol)

^k^ i-FGF23, Intact Fibroblast Growth Factor 23

^l^ c-FGF23, C-Terminal Fibroblast Growth Factor 23.

- Normal range: serum P were between (2.4 to 4.5 mg/dL); serum iCa (1.13–1.32 mmol/L); 25 (OH) D (8–42 ng/mL); 1,25 (OH)_2_ D_3_ (18–71 pg/mL); iPTH (15–65 pg/mL); *hs*-CRP0 (3 to 5 mg/L); albumin (3.4 to 5 g/dL) and Alkaline Phosphatase (35 to 104 U/L).

- To convert iCa from mEq/L to mmol/L, multiply by 0.5.

Inclusion in the longitudinal study was performed as follows: from the total of 150 patients, we included only patients that were on the same hemodialysis modality, HF-HD (n = 69). Twenty-six out of the 69 patients were excluded because they were on vitamin D or cinacalcet, which directly affects FGF23 values [32]. Patients on calcium containing phosphate binders (n = 14) were also excluded since they have been reported to have less effect than calcium-free phosphate binders in reducing FGF23 [33]. Patients who underwent kidney transplantation or died (n = 8) during follow-up were also excluded from the analysis. Finally, patients with poor nutrition and unstable medical condition or unwillingness to participate were also excluded. Thus, the final group included all patients treated with calcium-free phosphate binders (n = 21). Therefore, patients included in the longitudinal did not received calcium-based phosphate binders, paricalcitol nor cinacalcet before or during the study. Furthermore, they all received standard hemodialysis with a dialysis bath containing a calcium dialysate of 3mEq/L.

These 21 patients received standard dietary phosphate counseling as part of the routine dietary education provided to all of our dialysis patients. All patients received lanthanum carbonate or sevelamer to maintain serum phosphate below 4.5 mg/dl. The use of one or the other binder was based on patient and doctors’ preferences. The dose and duration of phosphate binders was modified by their nephrologist according to the serum phosphate concentration that was measured every two weeks. The dose of dialysis remained unchanged during the study. At the end of study, patients that achieved a serum phosphate below 4.5 mg/dL (P<4.5 mg/dL) were compared with those with serum phosphate >4.5 mg/dL. The target of a final serum phosphate <4.5 mg/dL was based on the median serum phosphate for each patient throughout the last 8 weeks of follow-up in agreement with the Spanish Society of Nephrology [34] and KDIGO recommendations [35].

The primary outcome in longitudinal analyses was to evaluate changes in serum FGF23 concentrations between the baseline and week-40 in patients with serum phosphate below or above 4.5 mg/dl. Other outcomes included modifications in serum concentration of phosphate, total calcium, ionized calcium, iPTH, *hs*-CRP, 25 (OH) D, and 1,25 (OH)_2_ D.

### Analysis of serum FGF23 levels in hemodialysis patients (n = 150)

A cross-sectional analysis was performed in 150 patients undergoing regular hemodialysis to determine serum concentrations of iFGF23 and cFGF23 and its relation with other parameters. The characteristics of the patients are shown in Table 1. From a total of 162 patients receiving hemodialysis in our facilities, we included a total of 150 that fulfilled the following inclusion criteria: on hemodialysis for more than three months and clinically stable without inflammatory disease, infection or malignancy. Patients with previous parathyroidectomy, inability to provide written informed consent (cognitive impairment) or unwillingness to donate blood samples for this study were not included.

### Blood samples, measurements, and assays

Plasma blood samples were obtained before the mid-week hemodialysis session. Serum phosphate and serum ionized calcium were measured within the next 6 hours after extraction. Aliquots for the measurement of iPTH, 25(OH) D, 1,25 (OH)_2_ D and both iFGF23 and cFGF23 were stored at -80°C so that all samples were measured the same day.

Blood ionized calcium was measured by the specific electrode (Ciba-Corning 800 series blood gas analyzer, Ciba-Corning, Essex, UK; reference range, 1.13–1.32 mmol/l). Serum phosphate was measured by spectrophotometry (Biosystems, Barcelona, Spain; reference range, 2.4–4.5 mg/dl). Vitamin-D levels 25(OH)D and 1,25 (OH)_2_D, were measured by RIA (Immunodiagnostic Systems, Boldon, UK; reference range 8–42 ng/ml and 18–71 pg/ml respectively). Serum PTH by IRMA (Coated bead, Scantibodies Laboratory, Santee, CA; reference range, 15–65 pg/ml). The cFGF23 molecule was measured in plasma by a two-site ELISA (Immutopics Inc., San Clemente, CA. Intra- and inter-assay CVs were 2.4% and 4.7%) and iFGF23 was measured by ELISA that recognized the intact active protein (Kainos Laboratories, Tokyo, Japan. Intra- and inter-assay CVs were 2.8% and 3.8%; reference range, 8.2–54.3 pg/ml). C-reactive protein was measured by the high-sensitivity assay (reference range, 0.3-5mg/l). Immunoturbidimetry (bromocresol purple) was used to measure serum albumin (reference range, 3.4–5 g/dl). Serum alkaline phosphatase was determined by the 4-nitro-phenyl phosphate assay (reference range, 35–104 U/l). The study was approved by the local institutional ethics committee (Comité de Ética de la Investigación de Córdoba; ref.2769, protocol FGF23_HD-2015). Written Informed consent was obtained from all patients. This research partially describes the results of protocol: NCT02697578.

### Statistical analyses

Data are expressed as mean ± standard deviation, (SD) or median (interquartile range, IQR) as appropriate. Categorical data are expressed as frequencies and proportions. Spearman correlation test was used to evaluate correlations between two variables. Nonparametric comparisons of group differences, such as Mann-Whitney and Kruskal–Wallis tests were used for quantitative variables where appropriate. On the contrary, parametric comparisons were performed using the one-way ANOVA with Bonferroni corrections for multiple comparisons.

Linear mixed models were used to compare longitudinal changes in serum phosphate, total calcium, iPTH, and hs-CRP between groups over time. iFGF23, cFGF23, serum ionized calcium, vitamin D, ferritin and TSAT from baseline to end of the study were analyzed using the paired T-test or the Wilcoxon signed-rank test as appropriate. Whether patients achieved or not the final serum phosphate target (final serum phosphate <4.5 mg/dl versus >4.5 mg/dl) was treated as fixed-effects. If the P-value comparing variables curves in patients with final serum phosphate <4.5 mg/dl versus those with final serum phosphate >4.5 mg/dl was significant, we also analyzed between-group differences in each of the variables. We also tested for interactions between serum phosphate and changes in *hs*-CRP (phosphate x change in *hs*-CRP) in relation to FGF23 serum levels.

Owing to the non-normal distribution, variables such as FGF23, iPTH, and *hs*-CRP were log-natural transformed. Patients were separated into two groups according to serum phosphate above or below its median concentration (4.35 mg/dL), and into tertiles of serum phosphate and contrasted in terms of all the other variables analyzed. Linear regression models (forward stepwise selection procedure) were used to examine factors that may affect the values of serum intact and c-terminal FGF23 in the overall population (n = 150). Variables that were statistically significant in the univariable analysis and others considered clinically relevant were included in the multivariable analysis. Then, additional multivariable regression analysis were performed in patients with serum phosphate levels below or above the median. Multicollinearity was corrected by restricting the condition index up to a maximum of 20, following Belsley`s criteria [36]. Additionally, the proportionate contribution of each predictor on the coefficient of determination (*R*^2^) was quantified by the calculation of the *Johnson`s Relative Weights (RWs)* [37,38]. *RW*s were calculate for all the variables included in the linear regression analysis although some of them were not statistically significant. *RW*s significance test were calculated as described elsewhere [39] and exclusively for variables statistically significant in linear regression models to enhance interpretability. *RW*s significance are reported as confidence intervals (CI); if zero is excluded in the CI, weights are significantly different. This statistical approach measures to what extent (expressed in percent) each variable contribute to regression equation in combination with other variables (*RWs*). Given the remarkable association between serum phosphate levels and FGF23, we finally stratified patients into different groups according to the respective median values of serum phosphate, iFGF23 and cFGF23. Four different groups were obtained combining high or low serum phosphate (P) with high or low iFGF23. Another four groups were obtained by combining high or low serum phosphate with high and low serum levels of cFGF23. According to values of iFGF23 the groups were: low P/low iFGF23, low P/high iFGF23, high P/low iFGF23, and high P/high iFGF23. In relation to cFGF23 the groups were: low P/low cFGF23, low P/high cFGF23, high P/low cFGF23, and high P/high cFGF23. SPSS statistics software 15.0 (Chicago, Illinois) and the GraphPad Prism 6.0c (GraphPad Software, La Jolla, CA) were used to perform all the statistical analysis. Two-sided *P* values <0.05 were considered statistically significant.

## Results

### Change in FGF23 levels after long-term control of serum phosphate concentration

Demographic and biochemical data of the 21 patients included in the prospective study are shown in Table 1. Also shown is the information of the 150 patients included in the cross-sectional study.

After 40 weeks, the 21 patients were separated into those that achieved the target serum phosphate concentration, less than 4.5 mg/dl, and those with a serum phosphate above 4.5 mg/dL. At baseline, the patients characteristics and parameters of mineral metabolism, including serum phosphate and FGF23 of both groups were similar independently of whether serum phosphate target was achieved during the study or not (Table 2). The only difference between the two groups at baseline was that of the serum levels of 25 (OH) D slightly lower in patients with serum phosphate >4.5 mg/dL at 40 weeks, [10.0 ng/mL (9.2–19.2) vs 8.0 ng/mL (6.3–9.8); *P* = 0.02, (Table 2). The Charlson comorbidity index was similar in both groups.

**Table 2:** Baseline clinical and biochemical characteristics of patients included in the longitudinal (40 weeks) study (*n* = 21). Patients are divided according to final serum phosphate, below or above 4.5 mg/dL. All patients were exposed to a dialysate containing 3 mEq/L of calcium and received HF-HD during the follow-up. Data are expressed as median and interquartile range.

Variable	Week 0 P <4.5 mg/dL (*n* = 12)	Week 0 P >4.5 mg/dL (*n* = 9)	P^*^
**Age (years)** ^**§**^	70.5 (62.5–80.5)	66.0 (56.0–73.0)	0.38
**Dialysis Vintage (months)** ^**a**^^,^ ^**§**^	55.8 (23.9–85.6)	53.7 (34.2–98.1)	0.80
**Dialysis Duration (min)** ^**§**^	245.0 (242.2–250.0)	245.0 (244.0–250.0)	0.55
**Charlson comorbidity Index** ^**§**^	4.0 (3–5.0)	3.0 (2.0–5–0)	0.21
**KtV** ^**§**^	1.9 (1.7–2.1)	2.1 (1,9–2.2)	0.11
**Kt (L/session)** ^**§**^	60.5 (58.4–63.7)	63.6 (56.5–67.5)	0.27
**Albumin (g/L)**	3.6 (3.3–3.8)	3.5 (3.3–3.9)	0.99
**Hb (g/L)** ^**b**^^,^ ^**§**^	11.5 (10.5–12.7)	11.2 (10.1–11.5)	0.19
**TSAT (%)** ^**c**^^,^ ^**§**^	30.0 (21.2–40.0)	29.0 (22.0–34.5)	0.50
**Ferritin (ng/ml)** ^**§**^	506.0 (393.0–700.0)	606.0 (312.0–833.5)	0.86
***hs-*CRP (mg/L)** ^**d**^^,^ ^**§**^	7.3 (3.3–11.0)	4.7 (2.7–8.8)	0.30
**Ca (mg/dL)** ^**e**^^,^ ^**§**^	8.88 (8.54–9.19)	8.93 (8.40–9.46)	0.91
**iCa (mEq/L)** ^**f**^^,^ ^**§**^	2.23 (2.15–2.24)	2.26 (2.16–2.31)	0.46
**P (mg/dL)** ^**g**^^,^ ^**§**^	4.1 (3.5–4.9)	3.8 (3.5–5.1)	0.80
**iPTH (pg/ml)** ^**h**^^,^ ^**§**^	287.3 (206.6–360.2)	236.8 (145.8–358.0)	0.86
**25 (OH) D (ng/ml)** ^**i**^^,^ ^**§**^	10.0 (9.2–19.2)	8.0 (6.3–9.8)	0.02
**1,25 (OH)**_**2**_ **D (pg/ml)** ^**j**^^,^ ^**§**^	9.2 (8.4–9.6)	8.6 (7.0–15.)	0.86
**iFGF23 (pg/ml)** ^**k**^^,^ ^**§**^	581.0 (491.2–886.0)	709.0 (179.0–1247.5	0.42
**cFGF23 (RU/ml)** ^**l**^^,^ ^**§**^	1034.0 (432.2–1368.7)	880.0 (610.5–2070.5)	0.65

^a^ Dialysis Vintage, Time since the initiation of dialysis

^b^ Hb, Hemoglobin levels

^c^ TSAT, Transferrin Saturation

^d^ *hs*-CRP, C Reactive Protein

^e^ Ca, Total serum Calcium

^f^ iCa, Serum Ionized Calcium

^g^ P, Serum Phosphate

^h^ iPTH, Intact Parathyroid Hormone

^i^ 25 (OH)D, 25 hydroxy vitamin D (calcidiol)

^j^ 1,25 (OH)_2_ D, 1,25 dihydroxy vitamin D (calcitriol)

^k^ i-FGF23, Intact Fibroblast Growth Factor 23

^l^ c-FGF23, C-Terminal Fibroblast Growth Factor 23.

^**§ **^Median and interquartile range (IQR)

* *P* value.

- The Mann-Whitney test was used as appropriate.

**Fig 1:**
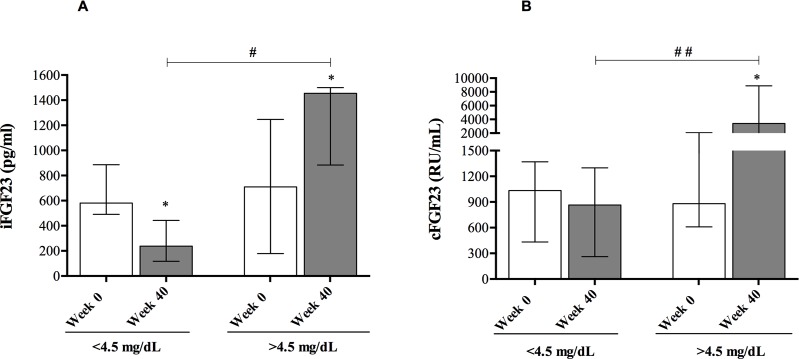
Serum concentrations of FGF23 at baseline and at week 40 in patients that achieved a serum phosphate concentration of <4.5 mg/dL and >4.5 mg/dL). **(A)** Change in iFGF23 concentration; **(B)** change in serum cFGF23 concentration. Bars represent median and interquartile range. Serum iFGF23 decreased from 581.0 pg/mL (491.2–886.0) to 238.5 pg/mL (116.7–443.5) [median percent change of 63.8% (-75.2–5.40) in patients that achieved the target of phosphate <4.5 mg/dL. In patients with serum phosphate >4.5 mg/dL, iFGF23 increased from 709.0 pg/mL (179.0–1247.5) to 1445.0 pg/mL (884.0–1500), [median percent change of 65.3% (9.1–368.1). cFGF23 did not decreased in those patients that achieved the target [median percent change -36.3 (-60.1–48.3). However, in patients with a final serum phosphate >4.5 mg/dl, it increased from 864.5 RU/mL (262.7–1299) to 3402.0 RU/ml (1899.0–8875), [median percent change of 206.9% (108.9–1056.3)]. * *P* <0.05 vs baseline. ^#^ *P* <0.05 versus same time different group. ^##^ *P*<0.001versus same time, different group.

The change in serum concentrations of iFGF23 and cFGF23 from baseline to the end of the study is shown in Fig 1. In patients with sustained control of serum phosphate, the serum concentration of iFGF23 decreased from 581.0 pg/mL (491.2–886.0) to 238.5 pg/mL (116.7–443.5), p = 0.03); the median percent change was 63.8% (IQR -75.2–5.40). On the contrary, in patients with serum phosphate >4.5 mg/dL, the concentration of iFGF23 increased from 709.0 pg/mL (179.0–1247.5) to 1445.0 pg/mL (884.0–1500), p = 0.03. [median percent change of 65.3% (9.1–368.1); Fig 1A]. cFGF23 did not decrease in patients with serum phosphate <4.5 mg/dL, but it did increase in patients with phosphate >4.5 mg/dL (Fig 1B).

**Fig 2:**
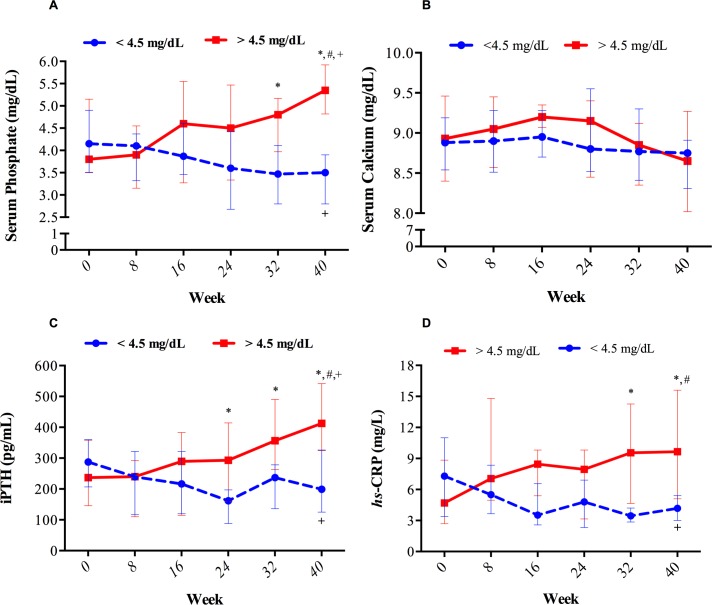
Change in parameters of mineral metabolism throughout the 40 weeks of follow-up in patients that completed the study with serum phosphate concentration above or below 4.5 mg/dL. In the group of patients with a final serum P<4.5 mg/dL, 54, 80, 80, 80, 100 and 100% of them had a ranged serum phosphate below the target at week 0, 8, 16, 24, 32 and 40 respectively. On the contrary, in the group with a final serum phosphate >4.5 mg/dl, 66, 77, 44, 44, 33 and 0% of the patients had serum phosphate levels below the study target along the study period. Dots represent median and whiskers represent IQR. * Between-group differences *P*<0.05. ^**+**^ Within-group differences *P*<0.05 for different groups. ^**#**^ *P*<0.001 for global comparison of curves.

Longitudinal trajectories of serum phosphate, total calcium, iPTH and *hs*-CRP are shown in Fig 2. By definition, the serum levels of phosphate were significantly higher in patients with a final serum phosphate >4.5 mg/dl as compared with those who did achieve the target (p<0.001 for global comparison) (Fig 2A). Between-group differences were reached at week 32 and 40 (p<0.05). The change in serum phosphate was associated with significant and concomitant change in iPTH despite the absence of changes in total serum calcium (P<0.01 for global comparison) (Fig 2B–2C). *hs*-CRP also increased in patients with final serum phosphate >4.5 mg/dL (P<0.01 for global comparison) (Fig 2D). Changes of the different parameters in patients within the same group showed similar results to those obtained by global comparisons. In patients achieving the target, serum phosphate (P<0.01), iPTH (P = 0.04), and hs-CRP (P<0.001) decreased significantly (Fig 2A–2D). By contrast, in the group of patients who did not achieve the target, serum phosphate and iPTH showed a significant increase (p<0.001 for both parameters). The values of *hs*-CRP showed an increasing trend that did not reach significance (P = 0.16). Nevertheless, at week 40, the levels of *hs*-CRP were significantly higher in patients who did not achieve the target than in those with final serum phosphate <4.5 mg/dl (p = 0.02). Between-group differences at the end of the study also showed significant differences in iPTH (p<0.01) (Fig 2C and Table 3). The total serum calcium and serum ionized calcium concentration did not change in both groups (Fig 2B and Table 3). No changes were observed from baseline to end of the study in dialysis duration, KtV, Kt, albumin, hemoglobin, TSAT, serum ferritin, 25 (OH) D or 1,25 (OH)_2_ D (Table 3). Phosphate x change in hs-CRP interaction was statistically significant (p for interaction = 0.03).

**Table 3:** The effect of long-term (40-weeks) control of serum phosphate (serum P <4.5 vs P >4.5 mg/dl) on different variables.

Variable	Week 40 P <4.5 mg/dL *n* = 12	Week 40 P >4.5 mg/dL *n* = 9	P^*^
**Dialysis Duration (mins)** ^**a**^^,^ ^**§**^	245.0 (243.0–247.5)	246.0 (243.5–249.5)	0.50
**KtV** ^**§**^	1.9 (1.7–2.0)	2.1 (1.8–2.3)	0.27
**Kt (L/session)** ^**§**^	61.4 (48.7–63.9)	64.0 (53.5–67.5)	0.24
**Albumin (g/L)**	3.6 (3.3–3.8)	3.5 (3.3–3.9)	0.88
**Hb (g/L)** ^**b**^^,^ ^**§**^	11.9 (11.2–13.4)	11.4 (10.7–12.0)	0.19
**TSAT (%)** ^**c**^^,^ ^**§**^	29.0 (23.7–46.0)	25.0 (21.5–31.5)	0.19
**Ferritin (ng/L)** ^**§**^	500.5 (268.7–827.0)	365.0 (173.5–655.5)	0.27
***hs-*CRP (mg/L)** ^**d**^^,^ ^**§**^	4.1 (3.0–5.4)	9.6 (5.2–15.)	0.02
**Ca (mg/dL)** ^**e**^^,^ ^**§**^	8.75 (8.31–8.91)	8.65 (8.02–9.27)	0.91
**iCa (mEq/L)** ^**f**^^**c6**^ ^**§**^	2.23 (2.20–2.25)	2.20 (2.05–2.32)	0.91
**P (mg/dL)** ^**g**^^,^ ^**§**^	3.5 (2.7–3.9)	5.3 (4.8–5.9)	<0.001
**iPTH (pg/ml)** ^**h**^^,^ ^**§**^	199.0 (125.1–326.4)	412.5 (325.0–541.2)	<0.01
**25 (OH) D (ng/ml)**^**i**^^,^ ^**§**^	11.8 (8.10–14.8)	8.29 (6.96–10.8)	0.09
**1,25 (OH)**_**2**_ **D (pg/ml)** ^**j**^^,^ ^**§**^	7.8 (7.2–8.8)	12.9 (5.2–14.4)	0.31
**iFGF23 (pg/ml)** ^**k**^^,^ ^**§**^	238.5 (131.7–443.5)	1455.0 (884.0–1500.0)	<0.01
**cFGF23 (RU/ml)**^**l**^^,^ ^**§**^	864.5 (262.7–1299.5)	3402.0 (1899.0–8875.0)	<0.001

^a^ Dialysis Duration; Effective duration of the dialysis session

^b^ Hb, Hemoglobin levels

^c^ TSAT, Transferrin Saturation

^d^ *hs*-CRP, C Reactive Protein

^e^ Ca, Total serum Calcium

^f^ iCa, Ionized Serum Calcium

^g^ P, Serum Phosphate

^h^ iPTH, Intact Parathyroid Hormone

^i^ 25 (OH)D, 25 hydroxy vitamin D (calcidiol)

^j^ 1,25 (OH)_2_ D, 1,25 dihydroxy vitamin D (calcitriol)

^k^ i-FGF23, Intact Fibroblast Growth Factor 23

^l^ c-FGF23, C-Terminal Fibroblast Growth Factor 23.

^§^ Median (IQR).

* *P* value

The IV iron preparation used in our study was the iron sucrose and it was prescribed to five out of 12 patients that reached a serum phosphate <4.5 mg/dl and 4 out of the 9 that ended with serum phosphate >4.5 mg/dL. The dose of IV iron was not different in both groups. There was no correlation between baseline ferritin level and the change in iFGF23 (r = -0.18, p = 0.42) or cFGF23 (r = 0.11, p = 0.62). No correlation was found between the baseline TSAT and the change in iFGF23 (r = -0.29, p = 0.18) or in cFGF23 (r = -0.18, p = 0.41). These finding persisted if the analysis was performed in the two groups separately. No association was found between EPO treatment and changes in either iFGF23 or cFGF23.

Regarding the use of phosphate binders, 66.7% (*n* = 14) of patients were on lanthanum carbonate at baseline, and 33.3% (*n* = 7) were on sevelamer. At the end of the study, all patients from the phosphate >4.5 mg/dL group were on both binders. Prescribed doses of phosphate binders during the study are shown in S1 Fig. As expected, patients with a worse phosphate control over the course of the study were prescribed higher doses of phosphate binders. Lanthanum carbonate was discontinued in one patient due to an excessive reduction in serum phosphate.

Further analysis, using the measurements from all patients revealed that the percent change in serum phosphate concentration was proportional to the percent change in both iFGF23 and cFGF23 molecules (Table 4). Percent change in serum phosphate also correlated with the change in iPTH and *hs*-CRP, which in turn was also correlated with changes in both FGF23 molecules (Table 4).

**Table 4:** Simple linear correlations between the percent change in serum phosphate concentration, both FGF23 molecules, serum iPTH concentration and *hs*-CRP (as an index of inflammation) in patients after 40 weeks of treatment.

Variable	iFGF23		cFGF23		Phosphate		iPTH	
	*r* ^*#*^	P*	*r* ^*#*^	P*	*r* ^*#*^	P*	*r* ^*#*^	P*
**cFGF23 (RU/mL)** ^**b**^	0.51	0.01	-	-	-	-	-	-
**Phosphate (mg/dL)** ^**c**^	0.78	<0.001	0.51	0.01	-	-	-	-
**iPTH (pg/mL)** ^**d**^	0.47	0.02	0.43	0.04	0.63	<0.01	-	-
***hs*-CRP (mg/L)** ^**e**^	0.49	0.02	0.61	<0.01	0.62	<0.01	0.63	<0.01

^a^ iFGF23, Intact Fibroblast Growth Factor 23

^b^ cFGF23, C-terminal Fibroblast Growth Factor

^c^ P, Serum Phosphate

^d^ iPTH, Intact Parathyroid Hormone

^e^ hs-CRP, High Sensitivity C-Reactive Protein.

The Spearman correlation test was used for all comparison.

^**#**^ Correlation Coefficient.

* *P*-value.

#### Analysis of iFGF23 and cFGF23 values in the entire population of hemodialysis patients

**Fig 3:**
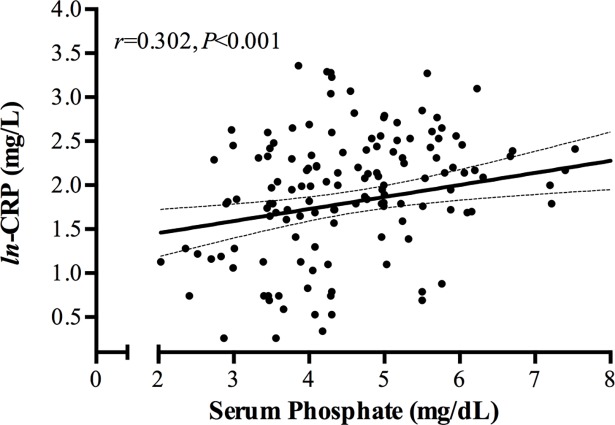
Scatter plot of serum phosphate levels and *ln*-CRP in the 150 patients on regular hemodialysis.

Further analyses of iFGF23 and cFGF23 levels were performed in our stable HD patients (*n* = 150) (Table 1). Both FGF23 molecules were significantly correlated with the serum concentration of phosphate, *hs-*CRP, age, and iPTH (Table 5 and S2 Fig). Also, the ln-cFGF23 showed a positive correlation with dialysis vintage and a negative correlation with 25 (OH) D (Table 5). Additionally, a significant positive correlation was observed between the serum concentration of serum phosphate and *hs*-CRP (Fig 3). The dose of erythropoietin did not correlate with serum levels of FGF23.

**Table 5:** Simple linear correlations between both FGF23 molecules and other demographic, clinical and CKD-MBD parameters (*n* = 150).

Variable	ln-iFGF23		ln-cFGF23	
	*r*	P*	*r*	P*
**ln-cFGF23** ^**a**^	0.70	<0.001	——	——
**Age (years)**	-0.25	<0.01	-0.26	<0.01
**Dialysis vintage (Months)**^**b**^	-0.02	0.74	0.21	<0.01
**Hb (g/L)** ^**c**^	0.05	0.49	-0.01	0.86
**TSAT (%)**^**d**^	-0.05	0.49	-0.04	0.58
**Ferritin (ng/L)**	-0.04	0.58	0.02	0.79
**ln-CRP (mg/L)** ^**e**^	0.33	<0.001	0.53	<0.001
**iCa (mEq/L)** ^**f**^	0.03	0.70	-0.14	0.07
**P (mg/dL)** ^**g**^	0.61	<0.001	0.55	<0.001
**ln-iPTH (pg/mL)**^**h**^	0.29	<0.001	0.29	<0.001
**25(OH)D (ng/mL)** ^**i**^	-0.06	0.44	-0.15	0.05
**1,25 (OH)**_**2**_ **D (pg/mL)** ^**j**^	0.03	0.67	0.06	0.46

^a^ ln-cFGF23, C-terminal Fibroblast Growth Factor

^b^ Dialysis Vintage, Time since the initiation of dialysis

^c^ Hb, Hemoglobin levels

^d^ TSAT, Transferrin Saturation

^e^ ln-CRP, C Reactive Protein

^f^ iCa, Serum Ionized Calcium

^g^ P, Serum Phosphate

^h^ ln-PTH, Intact Parathyroid Hormone

^i^ 25 (OH)D, 25 Hydroxyvitamin D (calcidiol)

^j^ 1,25 (OH)_2_D, 1,25 Dihydroxy vitamin D (calcitriol).

The Spearman correlation test was used for all comparison.

- To convert iCa in mEq/L to mmol/L, multiply by 0.5.

* *P*-value.

Table 6 shows clinical and biochemical data of patients according to serum phosphate tertiles contrasted in terms of the other parameters. Compared to the lowest tertile, patients in the highest phosphate tertile were more likely to be younger (p = 0.09) and showed higher serum *hs*-CRP (p = 0.01), iPTH (p<0.001), iFGF23 (p<0.01), and cFGF23 (p<0.001). Furthermore, serum ionized or total calcium was lower in patients with higher serum phosphate. Serum ferritin, TSAT, albumin and Charlson comorbidity index were comparable among groups (Table 6).

**Table 6:** Characteristics of the population included according to phosphate tertiles.

Variable	T1 (n = 49) <3.98 mg/dl	T2 (n = 52) 3.99–4.99 mg/dl	T3 (n = 49) >5 mg/dl	P
**Age (years; mean, s.d.)**	72.3 ± 15.7	66.2 ± 14.3	67.3 ± 14.5	0.09
**BMI (Mean, SEM) ^**a**^**	26.4 ± 6.4	26.1 ± 6.2	27.0 ± 4.8	0.73
**Gender (male; n, %)**	26 (53.1)	31 (59.6)	28 (57.1)	0.79
**Comorbidities**				
*Hypertension (%)*	43 (87.8)	42 (80.8)	40 (81.6)	0.59
*Diabetes (%)*	15 (30.6)	15 (28.8)	14 (28.6)	0.97
*Coronary artery disease (%)*	13 (26.5)	10 (19.2)	9 (18.4)	0.55
*Cerebrovascular disease (%)*	4 (8.2)	8 (15.4)	7 (14.3)	0.50
**Charlson comorbidity index**	4.0(2.0–5.0)	4.0 (2.0–5.0)	4.0 (2.0–5.0)	0.93
**Vascular Access**				
*AV Fistula*, *n (%)*	38 (77.6)	30 (57.7)	33 (67.3)	0.10
*Catheters*, *n (%)*	10 (20.4)	17 (32.7)	14 (28.6)	0.37
*Grafts*, *n (%)*	1 (2.0)	5 (9.6)	2 (4.1)	0.21
**Dialysate Calcium 3 mEq/L (n, %)**	44 (89.8)	44 (84.6)	43 (87.8)	0.73
**Dialysis technique** *HF-HD (n*, *%)* *OL-HDF (n*, *%)*	28 (44.9) 27 (55.1)	21 (40.4) 31 (59.6)	26 (53.1) 23 (46.9)	0.43
**Dialysis vintage (months)** ^**b**^^,^ ^**§**^	54.8 (16.9–84.5)	49.2 (16.4–76.8)	47.3 (18.6–86.5)	0.96
**Albumin (g/L) ^¶^**	3.6 ± 0.3	3.7 ± 0.3	3.7 ± 0.4	0.58
**Hb (g/L) ^c^^,^^¶^**	11.3 ± 1.2	11.1 ± 1.2	11.1 ± 1.4	0.58
**TSAT (%)^d^^,^^¶^**	28.7 ± 15.0	26.9 ± 9.90	28.8 ± 12.3	0.68
**Ferritin (ng/L) ^§^**	436.0 (343.0–714.5)	504.0 (331.5–742.7)	472.0 (287.5–849.5)	0.59
***hs-*CRP (mg/L) ^e^^,^^§^**	5.2 (2.6–8.2)	7.3 (4.2–10.9)	8.8 (5.8–12.4)	0.01
**Ca (mg/dL) ^e^^,^^¶^**	8.86 ± 0.47	8.81 ± 0.64	8.55 ± 0.77	0.03
**iCa (mEq/L) ^g^^,^^¶^**	2.21 ± 0.11	2.20 ± 0.16	2.13 ± 0.19	0.03
**P (mg/dl) ^h^^,^^§^**	3.4 (2.9–3.7)	4.3 (4.1–4.8)	5.7 (5.3–6.2)	<0.001
**Alkaline phosphatase (U/L) ^§^**	91.0 (74.5–115.0)	90.0 (69.0–121.7)	90.0 (71.0–124.0)	0.76
**iPTH (pg/ml) ^i^^,^^§^**	162.0 (104.0–268.9)	327.5 (167.0–429.7)	392.0 (204.5–672.5)	<0.001
**25(OH)D (ng/ml)^j^^,^^¶^**	10.7 ± 6.4	9.9 ± 5.0	9.0 ± 3.7	0.29
**1,25 (OH)_2_ D (pg/ml) ^k^^,^^¶^**	9.7 ± 6.2	10.5 ± 6.7	12.6 ± 6.7	0.08
**iFGF23 (pg/ml) ^l^^,^^§^**	158.0 (77.5–438.0)	502.5 (218.5–1167.7)	1030.0 (539.5–1754.0)	<0.01
**cFGF23 (RU/ml) ^m^^,^^§^**	471.0 (191.5–874.0)	959.5 (419.0–2049.7)	1561.0 (879.0–2652.5)	<0.001

^¶^ Mean ± Standard deviation (SD)

^§^ Median and Interquartile Range (IQR)

^a^ BMI, Body Mass Index

^b^ Dialysis Vintage, Time since the initiation of dialysis

^c^ Hb, Hemoglobin serum levels

^d^ TSAT, Transferrin Saturation

^e^ *hs*-CRP, C-Reactive Protein

^f^ Ca, total serum calcium

^g^ Ca, Ionized Serum Calcium

^h^ P, Serum Phosphate

^i^ iPTH, Intact Parathyroid Hormone

^j^ 25 (OH)D, 25 hydroxy vitamin D (calcidiol)

^k^ 1,25 (OH)_2_ D, 1,25 dihydroxy vitamin D (calcitriol)

^l^ iFGF23, Intact Fibroblast Growth Factor 23

^m^ cFGF23, C-Terminal Fibroblast Growth Factor 23.

- To convert iCa in mEq/L to mmol/L, multiply by 0.5.

- One-way ANOVA with Bonferroni corrections for multiple comparisons

In linear regression models, after adjustment for confounding variables, serum phosphate, *hs*-CRP, and also age, were independently correlated with both ln-iFGF23 and ln-cFGF23 levels (Table 7 and 8). Of note, serum ionized calcium, but not ln-iPTH, was also independently associated with high concentration of ln-iFGF23. Dialysis vintage was associated with high ln-cFGF23 levels, but not with elevated ln-iFGF23 (Table 7 and 8).

**Table 7:** Multivariable linear regression analysis showing the association between ln-iFGF23 as dependent variables and mineral metabolism parameters, inflammatory markers, and dialysis features, (*n* = 150).

Multivariable		ln-iFGF23	
**Variable**	**Beta**^**¶**^	**95% IC**	***P***
***Model 1***			
**ln-CRP**^**a**^	0.16	0.06–0.48	0.01
**iCa** ^**b**^	0.23	0.91–2.97	<0.001
**P** ^**c**^	0.65	0.51–0.76	<0.001
***Model 2***			
**Age**	-0.15	-0.02—-0.03	0.01
**ln-CRP** ^**a**^	0.18	0.10–0.51	<0.01
**iCa** ^**b**^	0.22	0.84–2.86	<0.001
**P** ^**c**^	0.61	0.48–0.72	<0.001
***Model 3***			
**ln-CRP**	0.16	0.06–0.48	<0.01
**iCa**	0.23	0.91–2.97	<0.001
**P** ^**c**^	0.65	0.51–0.76	<0.001

^a^ *hs*-CRP, C Reactive Protein

^b^ iCa, Serum Ionized Calcium

^c^ P, Serum Phosphate.

Model 1: adjusted for serum phosphate, ionized serum calcium, and hs-CRP. (R^2^ = 0.47)

Model 2: Adjusted for model 1 plus age, dialysis vintage, serum ferritin, iPTH, 25 (OH) D, and 1,25 (OH) _2_D. (R^2^ = 0.50)

Model 3: adjusted for model 1 plus calcium dialysate, the use of calcium-based binders, calcium-free binders, paricalcitol, cinacalcet and erythropoietin (R^2^ = 0.47)

^**¶ **^Standardized regression coefficients

**Table 8:** Multivariable linear regression analysis showing the association between ln-cFGF23 as dependent variables and mineral metabolism parameters, inflammatory markers, and dialysis features, (*n* = 150).

Multivariable		ln-cFGF23	
**Variable**	**Beta**^**¶**^	**95% IC**	***P***
***Model 1***			
**ln-CRP** ^**a**^	0.35	0.34–0.69	<0.001
**P** ^**b**^	0.50	0.32–0.52	<0.001
***Model 2***			
**Age**	-0.20	-0.02—-0.07	<0.001
**Dialysis Vintage** ^**c**^	0.23	0.003–0.01	<0.001
**ln-CRP** ^**a**^	0.37	0.37–0.70	<0.001
**P** ^**b**^	0.44	0.27–0.46	<0.001
***Model 3***			
**ln-CRP** ^**a**^	0.35	0.34–0.69	<0.001
**P** ^**b**^	0.50	0.32–0.52	<0.001

^a^ *hs*-CRP, C Reactive Protein

^b^ P, Serum Phosphate

^c^ Dialysis vintage, time since the initiation of dialysis.

Model 1: adjusted for serum phosphate, ionized serum calcium, and hs-CRP. (R^2^ = 0.46)

Model 2: adjusted for model 1 plus age, dialysis vintage, serum ferritin, iPTH, ferritin, 25 (OH) D, and 1,25 (OH) _2_D. (R^2^ = 0.54)

Model 3: adjusted for model 1 plus calcium dialysate, the use of calcium-based binders, calcium-free binders, paricalcitol, cinacalcet and erythropoietin (R^2^ = 0.46)

^**¶**^ Standardized regression coefficients

#### FGF23 levels in patients with adequate control of serum phosphate

Given that phosphate have been reported as one of the most important factors associated with the increase in FGF23, it is important to learn if in patients with acceptable control of serum phosphate, there are other variables that could be targeted to achieve a better control of the FGF23 molecules. Therefore, all patients (*n* = 150) were stratified according to whether the serum phosphate concentration was above or below the median (4.35 mg/L) (S1 Table). In patients with serum phosphate <4.35 mg/L, the ln-iFGF23 correlated with the serum concentration of phosphate and ionized calcium, and, the ln-cFGF23 correlated with serum levels of phosphate and *hs*-CRP (S2 Table). In patients with serum phosphate >4.35 mg/L, the ln-iFGF23 correlated with the serum concentration of phosphate, and *hs*-CRP, and did not correlate with serum ionized calcium (S2 Table). The ln-cFGF23 level correlated positively with serum phosphate, age, dialysis vintage, and serum *hs*-CRP. There was no correlation between values of ln-iPTH and FGF23 concentration (S2 Table). Finally, we also stratified patients into different groups according to the median values of serum phosphate, iFGF23 and cFGF23 (S3 table). Four different groups were obtained combining high or low serum phosphate (P) with high or low iFGF23; another four groups were obtained by combining high or low serum phosphate with high and low serum levels of cFGF23 (S3 table). Again, younger patients showed the highest serum levels of phosphate, iFGF23, and cFGF23. Subjects with high phosphate coinciding with high iFGF23 and cFGF23 showed the highest serum *hs*-CRP levels. The latter group of patients also had the highest iPTH serum levels. Interestingly, the group of patients with low phosphate/high cFGF23 also showed higher hs-CRP and longer dialysis vintage (S3 Table).

**Fig 4:**
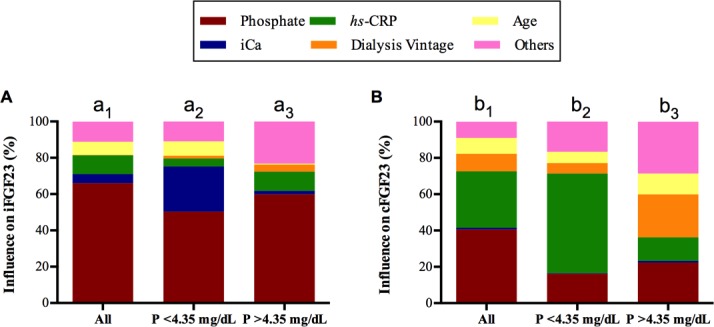
**The degree of influence (expressed in percent) of the various independent variables on the serum levels of iFGF23 (A) and cFGF23 (B).** The proportional contribution (Relative weights; *RW*s) of each of the independent variables on the serum levels of FGF23 were calculated. Statistical significance of *RW*s were assessed as described elsewhere [38,39]. Statistical significance is based on the values of confidence intervals; if zero is excluded from the confidence interval, the *RW* is significant. The *RW*s significance test was run only for variables that showed statistical significance in the linear regression models. The proportional contribution of serum iPTH, 25 (OH) D, 1,25 (OH) _2_ D, ferritin, calcium dialysate, the use of cinacalcet or paricalcitol, calcium-based, and calcium-free phosphate binders are grouped as “others” since their individual contribution was limited. S4 Table shows the detailed proportionate contribution of each variable for the entire population and separated according to phosphate levels below or above the median. Lowercase letters above columns identify different groups analyzed, a1-b1 overall population, a2-b2 patients with P<4.35 mg/dL and a3-b3 patients with P>4.35 mg/dL for iFGF23 and cFGF23 respectively. For iFGF23 **(A)** *RW*s of serum phosphate (CI for significance 0.24–0.47), ionized calcium [iCa] (CI for significance 0.001–0.07) and *hs*-CRP (CI for significance 0.01–0.10) were significantly different in the entire population (a_1)_. Moreover, *RW*s of serum phosphate was significantly greater than the *RW*s of iCa, *hs*-CRP and age *RW*s`. In patients with P<4.35 mg/dL (a_2)_, *RW*s of serum iCa (CI for significance 0.01–0.26) and phosphate (CI for significance 0.14–0.41) were significant as compared to the other variables. Interestingly, there was no difference between the *RW*s of phosphate and iCa in this group of patients (a_2_). In patients with phosphate above the median (P>4.35 mg/dL) [a_3_], only the *RW*s of *hs*-CRP (CI for significance 0.00–0.12) and phosphate (CI for significance 0.06–0.45) were significant. The *RW* of phosphate (59.9%) was significantly greater than that of the *RW*s of age (CI for significance -0.45—-0.04), *hs*-CRP (CI for significance -0.44—-0.02), and iCa (CI for significance -0.49—-0.07). **(B)** Regarding cFGF23, in the overall population (b_1_) serum phosphate remained to be the main contributor (40.6%). Together with phosphate (CI for significance 0.18–0.34), *RW*s of *hs*-CRP (CI for significance 0.10–0.26), dialysis vintage (CI for significance 0.01–0.14), and age (CI for significance 0.008–0.12) were also significant. *RW*s of phosphate and *hs*-CRP were not different (CI for significance -0.20–0.04). Contribution of *hs*-CRP was far more important than that of iCa (31.0 vs 1.0%); (b_1_). In the group of patients with P<4.35 mg/dL (b_2_), *hs*-CRP (CI for significance 0.09–0.40) and phosphate (CI for significance 0.02–0.20) were the two significant *RW*s. *hs*-CRP contributed far more than phosphate, age and dialysis vintage. Finally, in the group of patients with P>4.35 mg/dL (b_3_), dialysis vintage (CI for significance 0.05–0.32), *hs*-CRP (CI for significance 0.01–0.17), and serum phosphate (CI for significance 0.03–0.31) were the significant *RW*s. There were no differences between *RW*s of these three variables.

The degree of influence or relative weights (expressed in percent) of the various independent variables on serum levels of iFGF23 and cFGF23 is detailed in Fig 4 and S4 Table. In patients with serum phosphate <4.35 mg/dL, serum phosphate concentration contributed 50.3% to the high levels of iFGF23, serum ionized calcium concentration contributed 24.9%, and the *hs*-CRP only contributed 4.4%. By contrast, in patients with serum phosphate >4.35 mg/dL, although phosphate was still the most influential factor, *hs-*CRP contributed more than serum ionized calcium (10.6% vs. 1.9%) (Fig 4A). With regard to cFGF23, 81.3% of the relative weight was dependent on serum phosphate, *hs*-CRP, and dialysis vintage (Fig 4B and S4 Table). Of interest, high cFGF23 levels in patients with serum phosphate <4.35 mg/dL were more dependent on *hs*-CRP than serum ionized calcium.

## Discussion

The aim of the present study was to determine the effect of long-term control of serum phosphate on FGF23 levels. It was observed that reducing serum phosphate below the upper normal range (4.5 mg/dL) is associated with a decrease in iFGF23 although cFGF23 concentration did not change. By contrast, uncontrolled serum phosphate was associated with an increase in both iFGF23 and cFGF23. Interestingly, control of serum phosphate was also accompanied by a proportional reduction in serum levels of *hs*-CRP. Cross-sectional analysis of our population (*n* = 150) of hemodialysis patients confirmed a robust effect of serum phosphate concentrations on FGF23 levels. Even in patients with serum phosphate levels below the median, iFGF23 correlated with the prevailing serum phosphate, and also with high values of serum ionized calcium. Serum cFGF23 not only correlated with serum phosphate but also with serum *hs*-CRP instead of ionized calcium. Taken together, our results suggest that a reduction in serum FGF23 requires sustained control of serum phosphate. Moreover, a further reduction in FGF23 may be achieved by avoiding high serum calcium levels and maintaining low CRP levels.

A progressive reduction of serum phosphate was accompanied by a decrease in iPTH without changes in serum calcium which confirms that PTH is directly regulated by phosphate or through a reduction in skeletal resistance to PTH [40]. Also, *hs*-CRP levels were reduced in patients with serum phosphate <4.5 mg/dL, a finding not previously reported in HD patients. Both the reduction in iPTH and CRP levels may have contributed to the decrease in iFGF23 [9,16]. The opposite is true for patients unable to control serum phosphate. In these patients, the increase in PTH and CRP may have contributed to the elevation of iFGF23 and cFGF23. The magnitude of change in both iFGF23 and cFGF23 significantly correlated with the degree of change in serum phosphate concentration. In addition, the change in phosphate correlated with parallel changes in serum CRP levels.

We also examined the variables associated with the high circulating levels of iFGF23 and cFGF23 in HD patients. Our analysis revealed that iFGF23 and cFGF23 molecules correlated with each other. However, the independent variables that correlated with each of the FGF23 molecules were not totally the same, suggesting a differential regulation of each molecule. In the analysis by tertiles of phosphate and in linear regression models, younger age, high serum concentration of phosphate, and CRP were associated with high concentrations of both iFGF23 and cFGF23. However, while iFGF23 was also associated with a higher ionized calcium levels, cFGF23 was associated with the dialysis vintage.

In CKD patients, control of serum phosphate reduced FGF23 levels [23,24]. However, studies demonstrating such an effect on HD patients are limited. To assess the relationship between serum phosphate and FGF23 levels in HD patients, a subset of patients underwent strict control of serum phosphate that resulted in a parallel changes in FGF23. We observed a clear correlation between percent changes in phosphate and percent changes in iFGF23 and cFGF23. Moreover, the contribution of serum phosphate to the elevation of serum levels of iFGF23 was much greater than for cFGF23 (Fig 4). Thus, controlling serum phosphate below the upper normal limit was able to produce a substantial reduction in FGF23, but the levels still remained above normal. Evidently, the next question is once the serum phosphate is controlled, which other variables may further reduce the serum FGF23 levels. These other variables should be additional targets to reduce FGF23. In patients with serum phosphate within the normal range (<4.35mg/dL), cFGF23 was much more influenced by CRP whereas iFGF23 was more dependent on serum ionized calcium than CRP. Therefore, in patients with controlled serum phosphate, high serum calcium works against the reduction in iFGF23. The effect of serum calcium on FGF23 has been shown in animals [10–12,41]. Both serum calcium and CRP are modifiable variables that may affect FGF23 levels in patients with proper control of serum phosphate. PTH stimulates FGF23 production by activating the orphan nuclear receptor Nurr1 [42]. A feedback of FGF23 on PTH production may not be present in advanced hyperparathyroidism due to resistance to the action of FGF23 [8,43–46]. Simple linear correlation analysis showed that a high iPTH level was associated with elevated values of both FGF23 molecules. However, in multivariable analysis, PTH did not contribute significantly to the model predicting FGF23 values. The loss of its statistical power may be due to collinearity between the serum concentration of PTH and both serum P and calcium. Of interest, the reduction in FGF23 associated with cinacalcet may be potentiated by the decrease in serum calcium following the decline in serum phosphate and PTH [21]. This result is not surprising since calcimimetics decrease PTH and serum phosphate, and also reduces serum calcium, all of which have an effect in reducing FGF23 levels [21,47,48].

Regarding inflammation, our data show that high CRP levels correlated with cFGF23 and also but to a lesser extent with iFGF23 levels. In fact, the influence of CRP on cFGF23, as assessed by *RW*s was nearly 3-fold larger than that of CRP on iFGF23. Furthermore, patients with higher phosphate and higher iFGF23 and cFGF23 showed the highest serum *hs*-CRP levels suggesting that inflammation is likely associated with the increase in both FGF23 molecules. Recent studies have shown that there is a relationship between inflammation and FGF23 [15–17]. Indeed, FGF23 is likely to directly target FGFR4 in hepatocytes to promote the production of inflammatory cytokines in animal models of CKD [49]. Moreover, oral phosphate loading induces an increase in serum TNFα and IL-6 [50,51], an effect that was prevented by phosphate binders through the reduction in serum phosphate levels [52]. Thus, it is important to characterize this interrelationship since all of the three components, phosphate, inflammation, and FGF23 appear to be independently associated with mortality and cardiovascular disease in dialysis patients [20]. However, prospective studies designed to analyze the effect of serum phosphate control on inflammation in HD population are lacking. In the present study, we have observed that the reduction in serum phosphate was associated with a concomitant and commensurate reduction in CRP and FGF23. This fact suggests a tight relationship between the change in phosphate, CRP, and FGF23. Analysis of all patients revealed that the serum concentrations of phosphate and CRP were significantly correlated. However, we do not know if the reduction in serum phosphate decreased CRP directly or it is the reduction in FGF23 that caused the decrease in CRP. Thus, our study supports the notion that control of FGF23 via a reduction in serum phosphate may improve inflammatory status in hemodialysis patients. In fact, those patients with the lowest phosphate, iFGF23 and cFGF23 showed the lowest *hs*-CRP levels. Furthermore, we have observed, like others [53], that dialysis vintage was independently associated with higher cFGF23, which could be explained by long-term exposure to inflammation.

Age and gender have also been associated with elevated FGF23 [54]. However, this association remains controversial [55,56]. The increased FGF23 levels in younger patients may reflect higher protein and phosphate intake. In our study, age showed an inverse correlation with FGF23, which may be attributed to a more modest phosphate intake in older patients and decreased bone remodeling with older age.

Our study has limitations. As compared with other studies in dialysis patients we have included a relatively small number of patients. However, the simultaneous measurement of all blood parameters including intact and c-terminal FGF23 and the prospective evaluation of a subset of patients may have not only strengthened the results but also demonstrated for the first time in HD patients that the reduction in serum phosphate was associated with a reduction in CRP and FGF23. Moreover, all blood parameters were measured in the same sample including both FGF23 molecules, PTH, 25(OH) D, 1,25 (OH)_2_ D, *hs-*CRP, ionized calcium and serum phosphate concentration. In the present study, we did not find a significant effect of the type of treatment (P binders, vitamin D, calcimimetics) on FGF23 levels. It is likely that the effect of treatment is obscured by the concomitant changes in parameters that clearly affect FGF23 levels such as serum phosphate and calcium. A different study design would be required to analyze the effect of treatment on FGF23 levels.

In conclusion, different factors may independently influence intact and c-terminal FGF23. Control of serum phosphate is mandatory to reduce both iFGF23 and cFGF23. Reduction of inflammation is another relevant factor that influences the control of FGF23, but mainly the c-FGF23 molecule. It is likely that control of serum phosphate may also reduce inflammatory parameters possibly through a reduction in FGF23. In patients with controlled serum phosphate, a prevention of serum calcium elevation will further reduce iFGF23. Our data support the concept that serum phosphate, FGF23 levels, and inflammation are closely interrelated. These results may have clinical relevance because each of these parameters is associated with increased mortality.

## Supporting information

S1 Table Biochemical features of patients stratified by phosphate serum levels below (<4.35 mg/dL) or above the median (>4.35 mg/dL). (DOC)

S2 Table Multivariable linear regression analysis according to phosphate serum levels stratified as a binary variable. Determinants of high FGF23 serum levels in patients with phosphate below 4.35 mg/dL and above 4.35 mg/dL. A) iFGF23, B) cFGF23.
(DOC)

S3 Table Serum phosphate and FGF23 subgroups contrasted in terms of variables evaluated. Serum phosphate, iFGF23 and cFGF23 were stratified according to their median values and categorized into four different groups.
(DOC)

S4 Table Relative weights (*RWs*) of all variables clinically associated with an increase in FGF23. Relative contribution of each variable on serum iFGF23 or cFGF23 in the overall population (n = 150) and according to phosphate serum median (P<4.35 mg/dl or P>4.35 mg/dL). Some of the variables showed no statistical significance in linear regression analysis, however, its *RW* is shown.
(DOC)

S1 Fig Dose of prescribed Sevelamer and Lanthanum carbonate in patients with a good or poorer control of serum phosphate (<4.5 mg/dL vs >4.5 mg/dL). Doses changed according to phosphate modifications and patient preference. Dots represents median and whiskers 25th and 75th percentile. The number of patients under each treatment at each time-points are depicted under x-axis. After the 8th week, the number of patients who required both binders simultaneously increased. Between-group differences *P<0.05.
(DOC)

S2 Fig Correlations between FGF23, CKD-MBD parameters and ln-CRP. FGF23 and *hs*-CRP serum levels are expressed as log-natural transformed. Regression line is represented as a solid line and dashed line represents confidence interval (95% CI). Closed circles represent ln-iFGF23 and opened circles represent ln-cFGF23. Scatter plot of iFGF23 vs cFGF23 **(A),** iFGF23 and cFGF23 vs serum phosphate **(B-C)** and iFGF23 and cFGF23 vs hs-CRP levels **(D-E). **
(DOC)

S1 Dataset Compilation of data from patients included in the longitudinal analysis. (CSV)

S2 Dataset Compilation of data from patients included in the cross-sectional analysis. (CSV)
