# Supplementary material for: Phosphate control in reducing FGF23 levels in hemodialysis patients
Source: PLoS One. 2018 Aug 7;13(8):e0201537. doi: 10.1371/journal.pone.0201537 (PMC6080760; doi:10.1371/journal.pone.0201537)
Supplement: S1 Table — (DOC) [file pone.0201537.s001.doc]

**SUPPORTING INFORMATION**

**S1 Table. Biochemical features of patients stratified by phosphate serum levels below (<4.35 mg/dL) or above the median (>4.35 mg/dL).**

| **Variable** | **Phosphate**  **<4.35 mg/dL**  **n=75** | **Phosphate**  **>4.35 mg/dL**  **n=75** | ***P**** |
| --- | --- | --- | --- |
| **Age (years) ^§^** | 76.0 (61.0—84.0) | 67.0 (57.0—79.0) | <0.01 |
| **Dialysis Vintage (months) ^a, §^** | 53.7 (19.5—80.3) | 41.2 (13.5—85.1) | 0.38 |
| ***hs*-CRP (mg/L) ^b, §^** | 5.6 (2.3—9.2) | 8.5 (6.0—11.7) | <0.01 |
| **iCa (mEq/L) ^c^, ^§^** | 2.24 (2.14—2.30) | 2.14 (2.04—2.28) | 0.01 |
| **P (mg/dL) ^d, §^** | 3.7 (3.4—4.0) | 5.3 (4.9—5.9) | <0.001 |
| **iPTH (pg/mL) ^e, §^** | 211.0 (121.0—362.0) | 364.0 (201.0—560.5) | <0.001 |
| **25 (OH) D (ng/ml) ^f, §^** | 8.29 (7.0—11.1) | 8.1 (6.8—10.1) | 0.34 |
| **1,25 (OH)_2_ D (pg/ml) ^g, §^** | 11.0 (3.1—12.5) | 12.1 (8.6—16.1) | 0.01 |
| **iFGF23 (pg/ml) ^h, §^** | 223.0 (99.0—535.0) | 907.0 (447.0—1600.0) | <0.001 |
| **cFGF23 (RU/ml) ^i, §^** | 569.0 (242—965) | 1523.0 (795.0—2616.0) | <0.001 |

^§^ Median and Interquartile Range (IQR)

^a^ Dialysis Vintage, Time since the initiation of dialysis; ^b^ CRP, C Reactive Protein; ^c^ P, Serum Phosphate; ^d^ iCa, Serum Ionized Calcium; ^e^ iPTH, Intact Parathyroid Hormone; ^f^ 25 (OH)D, 25 Hydroxyvitamin D; ^g^ 1,25 (OH)_2_D, 1,25 Dihydroxyvitamin D; ^h^ i-FGF23, Intact Fibroblast Growth Factor 23; ^i^ c-FGF23, C-Terminal Fibroblast Growth Factor 23.

- To convert iCa in mEq/L to mmol/L, multiply by 0.5.

* *P* value
