# Supplementary material for: Phosphate control in reducing FGF23 levels in hemodialysis patients
Source: PLoS One. 2018 Aug 7;13(8):e0201537. doi: 10.1371/journal.pone.0201537 (PMC6080760; doi:10.1371/journal.pone.0201537)
Supplement: S2 Table — Determinants of high FGF23 serum levels in patients with phosphate below 4.35 mg/dL and above 4.35 mg/dL. A) iFGF23, B) cFGF23. (DOC) [file pone.0201537.s002.doc]

**SUPPORTING INFORMATION**

**S2 Table. Multivariable linear regression analysis according to phosphate serum levels stratified as a binary variable. Determinants of high FGF23 serum levels in patients with phosphate below 4.35 mg/dL and above 4.35 mg/dL. A) iFGF23, B) cFGF23.**

| ***A. ln-iFGF23*** | | | | | | |
| --- | --- | --- | --- | --- | --- | --- |
| **Multivariable** |  | **<4.35 mg/dL (n=75)** |  |  | ***>4.35 mg/dL (n=75)*** |  |
| **Variable** | **Beta^¶^** | **95% IC** | ***P*** | **Beta^¶^** | **95% IC** | ***P*** |
| ***Model 1*** |  |  |  |  |  |  |
| **ln-CRP (g/L) ^a^** | 0.07 | -0.15—0.37 | 0.39 | 0.21 | 0.02—0.65 | 0.03 |
| **iCa (mEq/L) ^b^** | 0.36 | 1.80—5.29 | <0.001 | 0.14 | -0.29—2.08 | 0.13 |
| **P (mg/dL) ^c^** | 0.57 | 0.86—1.65 | <0.001 | 0.52 | 0.31—0.67 | <0.001 |
| ***Model 2*** |  |  |  |  |  |  |
| **ln-CRP ^a^** | ----- | ----- | ----- | 0.21 | 0.02—0.65 | 0.03 |
| **iCa ^b^** | 0.36 | 1.80—5.29 | <0.001 | ----- | ----- | ----- |
| **P ^c^** | 0.57 | 0.86—1.65 | <0.001 | 0.52 | 0.31—0.67 | <0.001 |
| ***Model 3*** |  |  |  |  |  |  |
| **ln-CRP ^a^** | ----- | ----- | ----- | 0.21 | 0.02—0.65 | 0.03 |
| **iCa** | 0.36 | 1.80—5.29 | <0.001 | ----- | ----- | ----- |
| **P ^c^** | 0.57 | 0.86—1.65 | <0.001 | 0.52 | 0.31—0.67 | <0.001 |
| ***B.* ln-cFGF23** | | | | | | |
| **Multivariable** |  | **<4.35 mg/dL (n=75)** |  |  | ***>4.35 mg/dL (n=75)*** |  |
| **Variable** | **Beta^¶^** | **95% IC** | ***P*** | **Beta^¶^** | **95% IC** | ***P*** |
| ***Model 4*** |  |  |  |  |  |  |
| **ln-CRP ^a^** | 0.47 | 0.33—0.80 | *<0.001* | 0.27 | 0.10—0.68 | <0.001 |
| **P ^c^** | 0.26 | 0.12—0.85 | *<0.01* | 0.42 | 0.18—0.52 | <0.01 |
| ***Model 5*** |  |  |  |  |  |  |
| **ln-CRP ^a^** | 0.47 | 0.33—0.81 | *<0.001* | 0.28 | 0.15—0.67 | <0.01 |
| **P ^c^** | 0.26 | 0.11—0.83 | *0.01* | 0.32 | 0.11—0.42 | <0.01 |
| **Age (years)** | ----- | ----- | *-----* | -0.26 | -0.03— -0.06 | <0.01 |
| **Dialysis Vintage (months)** | ----- | ----- | *-----* | 0.36 | 0.004—0.01 | <0.001 |
| ***Model 6*** |  |  |  |  |  |  |
| **ln-CRP ^a^** | 0.47 | 0.33—0.81 | *<0.001* | 0.28 | 0.15—0.67 | <0.01 |
| **P ^c^** | 0.26 | 0.12—0.85 | *<0.01* | 0.32 | 0.11—0.42 | <0.01 |
| **Age (years)** | ----- | ----- | *-----* | -0.26 | -0.03— -0.006 | <0.01 |
| **Dialysis Vintage (months)** | ----- | ----- | *-----* | 0.36 | 0.004—0.01 | <0.001 |

^a^ *hs*-CRP, C Reactive Protein; ^b^ iCa, Serum Ionized Calcium; ^c^ P, Serum Phosphate; ^d^ Dialysis Vintage, Time since the initiation of dialysis.

Model 1: adjusted for serum phosphate, ionized serum calcium, and hs-CRP. ( R^2^=0.42 for the group with P<4.35 mg/dL and R^2^= 0.32 for the group with P>4.35 mg/dL)

Model 2: Adjusted for model 1 plus age, dialysis vintage, serum ferritin, iPTH, 25 (OH) D, and 1,25 (OH) _2_D. (R^2^=0.42 for the group with P<4.35 mg/dL)

Model 3: adjusted for model 1 plus calcium dialysate, the use of calcium-based phosphate binders, calcium-free phosphate binders, paricalcitol, cinacalcet and erythropoietin (R^2^=0.47 for the group with P<4.35 mg/dL and R^2^= 0.32 for the group with P>4.35 mg/dL)

Model 4: adjusted for serum phosphate, ionized serum calcium, and hs-CRP. ( R^2^=0.42 for the group with P<4.35 mg/dL and R^2^= 0.32 for the group with P>4.35 mg/dL)

Model 5: Adjusted for model 1 plus age, dialysis vintage, serum ferritin, iPTH, 25 (OH) D, and 1,25 (OH) _2_D. (R^2^=0.42 for the group with P<4.35 mg/dL)

Model 6: adjusted for model 1 plus calcium dialysate, the use of calcium-based phosphate binders, calcium-free phosphate binders, paricalcitol, cinacalcet and erythropoietin
