# Supplementary material for: Phosphate control in reducing FGF23 levels in hemodialysis patients
Source: PLoS One. 2018 Aug 7;13(8):e0201537. doi: 10.1371/journal.pone.0201537 (PMC6080760; doi:10.1371/journal.pone.0201537)
Supplement: S3 Table — Serum phosphate, iFGF23 and cFGF23 were stratified according to their median values and categorized into four different groups. (DOC) [file pone.0201537.s003.doc]

**S3 Table. Serum phosphate and FGF23 subgroups** contrasted in terms of variables evaluated. Serum phosphate and either iFGF23 and cFGF23 were stratified according to their median values and categorized into four different groups.

|  | **Phosphate and iFGF23 subgroups** | | | | | **Phosphate and cFGF23 subgroups** | | | | |
| --- | --- | --- | --- | --- | --- | --- | --- | --- | --- | --- |
| **Variable** | **Low P/**  **Low iFGF23**  **(n=55)** | **Low P/**  **High iFGF23**  **(n=22)** | **High P/**  **Low iFGF23**  **(n=20)** | **High P/**  **High iFGF23**  **(n=53)** | **P*** | **Low P/**  **Low cFGF23**  **(n=53)** | **Low P/**  **High cFGF23**  **(n=22)** | **High P/**  **Low cFGF23**  **(n=22)** | **High P/**  **High cFGF23**  **(n=53)** | **P*** |
| **Age (years; mean, s.d.)** | 74.8 ± 12.8 | 64.0 ± 16.7 | 66.9 ± 15.2 | 65.5 ± 14.2 | <0.001 | 72.4 ± 15.5 | 68.2 ± 14.5 | 71.4 ± 13.6 | 63.7 ± 14.0 | <0.01 |
| **BMI (Mean, SEM) ^a, ¶^** | 26.3 ± 5.96 | 27.2 ± 8.27 | 26.6 ± 4.34 | 26.3 ± 5.1 | 0.93 | 25.8 ± 6.45 | 28.5 ± 7.14 | 26.8 ± 3.43 | 28.5 ± 7.14 | 0.36 |
| **Gender (male; n, %)** | 30 (54.5) | 11 (50.0) | 14 (70.0) | 30 (56.6) | 0.58 | 29 (54.7) | 10 (45.5) | 18 (81.8) | 28 (52.8) | 0.07 |
| **Charlson comorbidity index** | 3.0 (2.0—5.0) | 4.0 (3.0—4.2) | 4.0 (2.2—5.0) | 3.0 (2.0—5.0) | 0.46 | 3.0 (2.0—5.0) | 4.0 (2.0—5.0) | 4.0 (2.7—5.0) | 3.0 (2.0—5.0) | 0.70 |
| **Dialysate Calcium 3 mEq/L (n, %)** | 47 (85.5) | 20 (90.9) | 18 (90.0) | 46 (86.8) | 0.90 | 46 (86.8) | 20 (90.9) | 21 (95.5) | 44 (83.0) | 0.48 |
| **Dialysis vintage (months) ^b, §^** | 54.8  (27.3—80.2) | 40.3  (15.4—85.8) | 35.7  (6.6—67.3) | 47.3  (15.5—86.8) | 0.35 | 53.1  (18.7—79.5) | 59.6  (21.6—85.8) | 29.6  (6.8—48.6) | 52.9  (15.5-89.9) | 0.06 |
| **Dialysis Duration (min) ^c, ¶^** | 246.8 ± 14.3 | 248.7 ± 8.9 | 243.4 ± 15.3 | 247.3 ± 12.1 | 0.13 | 247 ± 12.5 | 249.6± 7.8 | 241.4 ± 18.7 | 247.2 ± 12.0 | 0.08 |
| **Albumin (g/L)** | 3.6 ± 0.3 | 3.6 ± 0.2 | 3.8 ± 0.2 | 3.6 ± 0.4 | 0.12 | 3.6 ± 0.4 | 3.6 ± 0.2 | 3.8 ± 0.3 | 3.6 ± 0.3 | 0.13 |
| **Hb (g/L) ^d, ¶^** | 11.1 ± 1.2 | 11.4 ± 1.1 | 11.1 ± 0.9 | 11.1 ± 1.4 | 0.91 | 11.3 ± 1.3 | 11.1 ± 1.0 | 11.3 ± 1.0 | 11.0 ± 1.4 | 0.73 |
| **TSAT (%)^e, §^** | 25.0  (20.0—35.0) | 25.5  (22.0—31.0) | 30.5  (20.0—39.2) | 26.0  (20.0—35.0) | 0.69 | 25.0  (20-0—30.5) | 27.5  (22.7—39.5) | 32.5  (23.7—41.0) | 26.0  (19.0—32.0) | 0.03 |
| **Ferritin (ng/dl) ^§^** | 466.0  (337.0—742.0) | 453.0  (348.0—652.2) | 383.0  (277.4—690.2) | 545.0  (341.5—806.5) | 0.47 | 440.0  (309.0—740.5) | 471.5  (368.7—552.2) | 612.0  (303.7—869.2) | 436.0  (305.0—806.5) | 0.82 |
| ***hs-*CRP (mg/L) ^f, §^** | 5.4 (2.80-8.80) | 6.5 (2.2-14.2) | 6.8 (5.0-8.50) | 9.50 (6.30-12.6) | <0.001 | 3.6 (2.1-7.1) | 10.0 (6.9-16.3) | 6.7 (5.42-8.2) | 10.1 (6.3-12.7) | <0.001 |
| **Ca (mg/dL) ^g, ¶^** | 8.82 ± 0.49 | 9.01 ± 0.53 | 8.63 ± 0.83 | 8.60 ± 0.72 | 0.03 | 8.83 ± 0.51 | 8.95 ± 0.48 | 8.86 ± 0.80 | 8.51 ± 0.72 | 0.02 |
| **iCa (mEq/L) ^h, ¶^** | 2.20 ± 0.12 | 2.25 ± 0.13 | 2.15 ± 0.20 | 2.14 ± 0.18 | 0.03 | 2.20 ± 0.12 | 2.23 ± 0.12 | 2.21 ± 0.20 | 2.12 ± 0.18 | 0.02 |
| **P (mg/dl) ^i, ¶^** | 3.5 ± 0.6 | 3.9 ± 0.3 | 5.2 ± 0.5 | 5.8 ± 1.3 | <0.001 | 3.5 ± 0.5 | 3.8 ± 0.3 | 5.1 ± 0.5 | 5.8 ± 1.3 | <0.001 |
| **Alkaline phosphatase (U/L) ^§^** | 91.0  (71.0—119) | 96.0  (73.5—110.5) | 92.0  (68.7—122.7) | 86.0  (70.5—127.5) | 0.99 | 96.0  (74.5—120.0) | 81.5  (68.0—107.7) | 80.0  (67.5—112.7) | 91.0  (71.0—130.5) | 0.31 |
| **iPTH (pg/ml) ^j, §^** | 167.0  (107.0—359.0) | 290.0  (157.0—382.0) | 329.0  (188.2—425.0) | 400.0  (201.5—689.5) | <0.001 | 170.0  (105.5—360.5) | 255.0  (126.0—370.2) | 274.0  (206.5—394.0) | 418.0  (191.0—689.5) | <0.001 |
| **25(OH)D (ng/ml) ^k, §^** | 8.4  (7.5—11.6) | 7.4  (6.5—10.0) | 8.4  (7.0—10.0) | 8.0  (6.7—11.3) | 0.69 | 9.1  (7.5—12.2) | 7.8  (5.0—10.0) | 8.1  (6.2—9.9) | 8.0  (6.8—10.7) | 0.69 |
| **1,25 (OH)_2_ D (pg/ml) ^l, §^** | 11.0  (4.0—12.2) | 11.3  (2.6—13.0) | 13.2  (5.3—18.4) | 12.0  (9.0—16.3) | 0.05 | 11.0  (3.3—13.6) | 6.5  (2.7—12.2) | 11.4  (5.0—16.7) | 12.3  (9.0—16.0) | 0.17 |
| **iFGF23 (pg/ml) ^m, §^** | 148.0  (75.0-262.0) | 1072.0  (620.01466.2) | 282.5  (169.2-412.7) | 1357.0  (884.0-2424.0) | <0.001 | 158.0  (82.5—414.0) | 574.5  (281.2—1358.7) | 415.5  (223.2—580.0) | 1179.0  (788.5—2424.0) | <0.001 |
| **cFGF23 (RU/ml) ^n, §^** | 401.0  (183.0—780.0) | 969.5  (596.2--1797.7) | 665.5  (404.5-1050.0) | 1995.0  (1188.0--3444.5) | <0.001 | 340.0  (179.5—614.0) | 1477.0  (1145-7—2128.5) | 575.5  (387.7—727.0) | 2066.7  (1400.5—4135.5) | <0.001 |

^¶^ Mean and Standard deviation (DS)

^§^ Median and Interquartile Range (IQR)

^a^ BMI, Body Mass Index; ^b^ Dialysis Vintage, Time since the initiation of dialysis; ^c^ Dialysis duration, Time of duration of the dialysis session; ^d^ Hb, Hemoglobin; ^e^ TSAT, Transferrin Saturation; ^f^ *hs*-CRP, C Reactive Protein; ^g^ Ca, Total serum calcium; ^h^ iCa, Ionized Serum Calcium; ^i^ P, Serum Phosphate; ^j^ PTH, Intact Parathyroid Hormone; ^k^ 25 (OH)D, 25 hydroxy vitamin D (calcidiol); ^l^ 1,25 (OH)_2_ D, 1,25 dihydroxy vitamin D (calcitriol); ^m^ i-FGF23, Intact Fibroblast Growth Factor 23; ^n^ c-FGF23, C-Terminal Fibroblast Growth Factor 23.

- Serum normal values of serum P were between 2.4 to 4.5 mg/dL, 1.13 – 1.32 mmol/L for iCa, 8 – 42 ng/mL for 25 (OH) D, 18 – 71 pg/mL for 1,25 (OH)_2_ D and 15 – 65 pg/mL for PTH. *hs*-CRP normal range was within 0.3 to 5 mg/L. Albumin´s normal range was from 3.4 to 5 g/dL and from 35 to 104 U/L for Alkaline Phosphatase.

- To convert iCa in mEq/L to mmol/L, multiply by 0.5.

* The Kruskal-Wallis test was used to evaluate comparisons between different subgroups.
