# Supplementary material for: Phosphate control in reducing FGF23 levels in hemodialysis patients
Source: PLoS One. 2018 Aug 7;13(8):e0201537. doi: 10.1371/journal.pone.0201537 (PMC6080760; doi:10.1371/journal.pone.0201537)
Supplement: S4 Table — Relative contribution of each variable on serum iFGF23 or cFGF23 in the overall population (n = 150) and according to phosphate serum median (P<4.35 mg/dl or P>4.35 mg/dL). Some of the variables showed no statistical significance in linear regression analysis, however, its RW is shown. (DOC) [file pone.0201537.s004.doc]

**S4 Table. Relative weights (*RWs*) of all variables clinically associated with an increase in FGF23. Relative contribution of each variable on serum iFGF23 or cFGF23 in the overall population (n=150) and according to phosphate serum median (<4.35 mg/dl or P>4.35 mg/dL). Some of the variables showed no statistical significance in linear regression analysis, however, its *RW* is shown.**

|  | **iFGF23** | | | **cFGF23** | | |
| --- | --- | --- | --- | --- | --- | --- |
|  | **All  (*n*=150)** | **P<4.35 mg/dL  (*n*=75)** | **P>4.35 mg/dL  (*n*=75)** | **All  (*n*=150)** | **P<4.35 mg/dL  (*n*=75)** | **P>4.35 mg/dL  (*n*=75)** |
| **Variable** | ***RW***** | ***RW* **** | ***RW* **** | ***RW***** | ***RW* **** | ***RW* **** |
| **Age (Years)** | 7.2 | 7.8 | 0.5 | 8.7 | 6.3 | 11.5 |
| **Dialysis Vintage (Months)^a^** | 0.2 | 1.6 | 3.9 | 9.7 | 5.7 | 23.7 |
| **Calcium Dialysate  *2.5 mEq/L  3 mEq/L*** | ref 0.1 | 0.2 | 3.9 | 0.7 | 0.9 | 5.6 |
| **ln-CRP ^b^** | 10.4 | 4.4 | 10.6 | 31.0 | 54.9 | 12.9 |
| **iCa ^c^** | 5.0 | 24.9 | 1.9 | 1.0 | 0.3 | 1.0 |
| **P ^d^** | 66.0 | 50.3 | 59.9 | 40.6 | 16.2 | 22.3 |
| **ln-iPTH ^e^** | 3.9 | 1.6 | 0.4 | 3.1 | 0.7 | 1.5 |
| **25 (OH) D ^f^** | 0.6 | 1.2 | 2.3 | 0.6 | 2.4 | 2.9 |
| **1,25 (OH)_2_ D ^g^** | 0.8 | 0.4 | 2.4 | 0.5 | 0.5 | 2.6 |
| **Ferritin** | 0.3 | 0.3 | 0.5 | 0.2 | 2.6 | 5.3 |
| **Cinacalcet  *No  Yes*** | ref 0.3 | 2.6 | 0.3 | 1.5 | 0.4 | 1.1 |
| **Paricalcitol  *No  Yes*** | ref 1.4 | 2.4 | 1.5 | 0.5 | 5.5 | 4.7 |
| **Calcium-based Binders  *No  Yes*** | ref 2.2 | 1.0 | 8.0 | 0.5 | 2.1 | 1.5 |
| **Calcium-free Binders  *No  Yes*** | ref 0.8 | 0.5 | 2.9 | 0.6 | 0.2 | 0.4 |
| **Erythropoietin  *No  Yes*** | ref 0.8 | 0.8 | 1.0 | 0.8 | 1.3 | 3.0 |

^a^ Dialysis Vintage, Time since the initiation of dialysis; ^b^ hs-CRP, C-Reactive Protein; ^c^ iCa, Serum Ionized Calcium; ^d^ P, Serum Phosphate; ^e^ ln-PTH, Parathyroid Hormone; ^f^ 25 (OH) D, 25 Hydroxyvitamin D; ^g^ 1,25 (OH)_2_ D, 1,25 Dihydroxyvitamin D.
***RW*: Relative Weight (Influence of each variable on regression equation in combination with other variables included in the model. Data is expressed as percentage of contribution to the multiple R)
