# Supplementary material for: Phosphate control in reducing FGF23 levels in hemodialysis patients
Source: PLoS One. 2018 Aug 7;13(8):e0201537. doi: 10.1371/journal.pone.0201537 (PMC6080760; doi:10.1371/journal.pone.0201537)
Supplement: S1 Fig — Doses changed according to phosphate modifications and patient preference. Dots represents median and whiskers 25th and 75th percentile. The number of patients under each treatment at each time-points are depicted under x-axis. After the 8th week, the number of patients who required both binders simultaneously increased. Between-group differences *P<0.05. (DOC) [file pone.0201537.s005.doc]

**SUPPORTING INFORMATION**

**S1 Fig. Dose of prescribed Sevelamer and Lanthanum carbonate in patients with a good or poorer control of serum phosphate (<4.5 mg/dL vs >4.5 mg/dL).** Doses changed according to phosphate modifications and patient preferences. Dots represents median and whiskers 25th and 75th percentile. The number of patients under each treatment at each time-points are depicted under x-axis. After the 8th week, the number of patients who required both binders simultaneously increased. Between-group differences **P*<0.05.
