# Supplementary material for: Phosphate control in reducing FGF23 levels in hemodialysis patients
Source: PLoS One. 2018 Aug 7;13(8):e0201537. doi: 10.1371/journal.pone.0201537 (PMC6080760; doi:10.1371/journal.pone.0201537)

**Supporting Information**

**S2 Fig. Correlations between FGF23, CKD-MBD parameters and ln-CRP.** FGF23 and CRP serum levels are expressed as log-natural transformed. Regression line is represented as a solid line and dashed line represents confidence interval (95% CI). Closed circles represent ln-iFGF23 and opened circles represent ln-cFGF23. Scatter plot of i-FGF23 vs c-FGF23 **(A),** i-FGF23 and c-FGF23 vs serum phosphate **(B-C)** and i-FGF23 and c-FGF23 vs CRP levels **(D-E).**


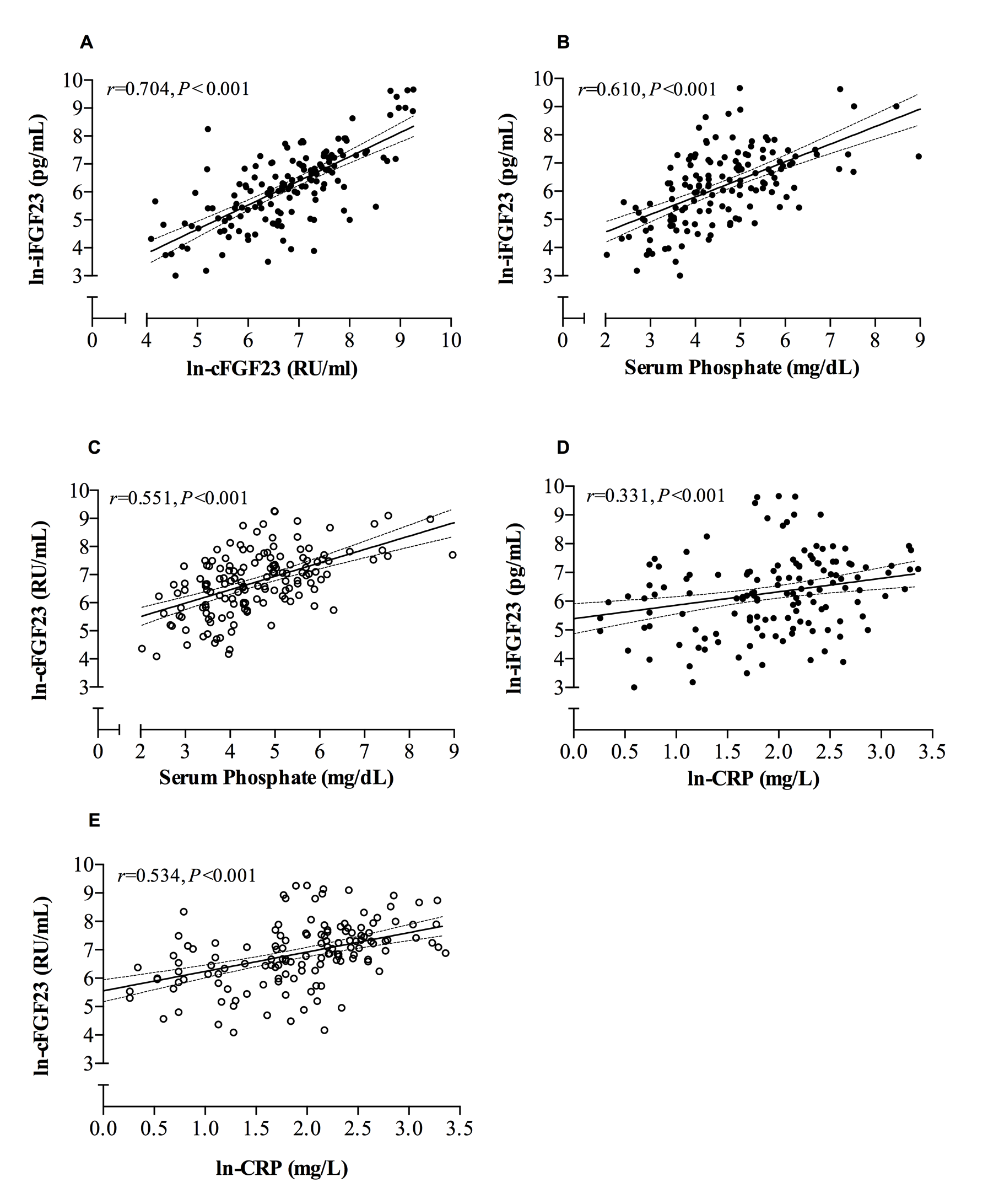

Supplement: S2 Fig — FGF23 and hs-CRP serum levels are expressed as log-natural transformed. Regression line is represented as a solid line and dashed line represents confidence interval (95% CI). Closed circles represent ln-iFGF23 and opened circles represent ln-cFGF23. Scatter plot of iFGF23 vs cFGF23 (A), iFGF23 and cFGF23 vs serum phosphate (B-C) and iFGF23 and cFGF23 vs hs-CRP levels (D-E). (DOC) [file pone.0201537.s006.doc]
